# UPLC-MS metabolite profiling and antioxidant efficacy of *Neopestalotiopsisclavispora* isolated from *Oroxylum indicum* (L.) Kurz Stem Bark

**DOI:** 10.1371/journal.pone.0357595

**Published:** 2026-09-09

**Authors:** Sushma Swaroopa, Amshumala Bhoothakallu Lolajaksha, Tamizh Selvan Gnanasekaran, Pavan Gollapalli, Vishakh Radhakrishna Kedilaya, Sharmila Kameyanda Poonacha

**Affiliations:** 1 Nitte (Deemed to be University), KS Hegde Medical Academy (KSHEMA), Central Research Laboratory, Mangaluru, Karnataka, India; 2 Department of Bioinformatics and Biostatistics, Nitte (Deemed to be University), Nitte University Centre for Science Education and Research (NUCSER), Paneer Campus, Mangaluru, Karnataka, India; 3 Nitte (Deemed to be University), KS Hegde Medical Academy (KSHEMA), Center for Bioinformatics, Mangaluru, Karnataka, India; Jadavpur University, INDIA

## Abstract

Endophytic fungi represent a valuable source of bioactive compounds with diverse pharmacological properties. In this study, we explored the antioxidant potential of secondary metabolites produced by *Neopestalotiopsis clavispora (N.clavispora)*, an endophytic fungus isolated from *Oroxylum indicum Kurz stem bark. N.clavispora* extract was subjected to UPLC-MS analysis to profile its bioactive components, and its antioxidant capacity was assessed using the DPPH (1,1-diphenyl-2-picrylhydrazyl) free radical scavenging assay. For *in vivo* evaluation, Swiss albino mice were administered with 100 mg/kg bodyweight dose of extract for five consecutive days, followed by exposure to 2 Gy of gamma radiation at a dose rate of 3.58 Gy/min. Subsequent measurements of superoxide dismutase (SOD) activity in liver homogenate were conducted to assess antioxidant enzyme response. The data obtained from UPLC-MS, showed, cimicifoetiside B (identified by UNIFI database matching; 44% relative abundance) as the predominant metabolite. *N. clavispora* extract showed moderate DPPH scavenging (IC₅₀ 82.86 μg/mL vs ascorbic acid 19.01 μg/mL). Molecular docking using cimicifoetiside B against the antioxidant enzyme Superoxide dismutase (SOD), revealing a docking score of −4.96 kcal/mol with 1 hydrogen bond. Molecular dynamics simulations confirmed the interaction with a binding energy of –12.36 ± 02.32 kcal/mol. Binding affinities and molecular interaction profiles indicated possible association with SOD. *In vivo*, *N.clavispora* extract at 100 mg/kg body weight/day yielded SOD activity of 1.98 ± 0.38 vs. radiation group 0.78 ± 0.13. These results suggest *N.clavispora* may have antioxidant related activity under the conditions tested, supporting further *in vitro* and *in vivo* studies.

## Introduction

Reactive oxygen species (ROS), are chemically reactive entities produced in living organisms as a result of electron transfer processes in which molecular oxygen serves as the acceptor. ROS include oxygen radicals such as the hydroxyl radical (^•^ OH), peroxyl radical (ROO^•^), the superoxide anion radical (O_2_^• -^) or non-radical compounds such as hydrogen peroxide (H_2_O_2_) or singlet oxygen (^1^ O_2_). When the production of ROS exceeds the capacity of antioxidant systems to neutralize them, it creates oxidative stress that alters cellular biomolecules, including nucleic acids, proteins, and membrane lipids [1]. Research has shown that oxidative stress triggered by reactive oxygen species contributes not only to the ageing process but also to the onset and progression of numerous human disorders, including cancer, cardiovascular complications (such as atherosclerosis, ischemic heart disease, hypertension, cardiomyopathies, cardiac hypertrophy, and heart failure), type 2 diabetes, cataracts, rheumatoid arthritis, and various neurodegenerative conditions. [2]. Antioxidants are essential components of the body’s defense system, helping to counteract the damaging effects of reactive oxygen species. In a healthy state, this protection depends on a well-regulated balance between oxidants and antioxidant defenses. Since aerobic metabolism continuously produces free radicals, their harmful effects must be offset by an equivalent rate of antioxidant activity. These protective molecules, which may act through enzymatic pathways or as non-enzymatic agents, work by preventing free radical formation, neutralizing them, or repairing the molecular damage they cause. Both endogenous and externally derived antioxidants contribute to safeguarding cells from oxidative injury and associated chronic diseases [3,4].

Endophytes are microorganisms that reside within plant tissues without causing harm and have been identified as significant contributors to plant antioxidant defenses. For instance, a study on *Pestalotiopsis neglecta*, an endophytic fungus isolated from *Ziziphus spina-christi*, revealed that its extracts exhibited substantial antioxidant activity, attributed to the presence of phenols, flavonoids, and terpenoids.These compounds effectively neutralized free radicals and protected DNA from oxidative damage, highlighting the potential of endophytes in enhancing plant resilience through natural antioxidant production [5]. *Neopestalotiopsis* species are endophytic, plant-pathogenic, or saprobic fungi found on living plants and is widely distributed throughout tropical and temperate regions [6]. Studies investigating the secondary metabolites produced by *N. clavispora* remain limited. This fungus is capable of producing a wide variety of metabolites, particularly belonging to the polyketide and terpenoid classes, many of which exhibit significant biological activity [7,8]. Available information on the bioactive compounds produced by *N. clavispora* remains limited. Research into the secondary metabolites of endophytic fungi not only uncovers novel bioactive substances but also reveals a wide range of biological activities, such as phytotoxicity, antibacterial, antifungal, anti-inflammatory, and antitumor effects. These findings present new opportunities for discovering promising candidates in agricultural and pharmaceutical drug development [9].

The current investigation sequentially integrates UPLC-MS profiling of *N. clavispora* extract to identify bioactive components, *in vitro* DPPH antioxidant assay, *in vivo* evaluation of hepatic SOD activity a key antioxidant biomarker in mice subjected to 2 Gy γ-radiation as an oxidative stress model, and *in silico* docking of predominant metabolite cimicifoetiside B against SOD (PDB: 5VF9). SOD serves dual roles here: first as our experimentally measured biomarker of systemic antioxidant response, and second as the primary docking target due to its enzymatic role dismutatingsuperoxi radiation-induced de radicals (O_2_^-^) ahead of downstream enzymes catalase (H_2_O_2_) and GPx (peroxides), providing a basis for exploring possible antioxidant-related and radioprotective effects of endophyte-derived compounds.

## Materials and methods

### Plant identification

The stem bark of *Oroxylum indicum* (family: Bignoniaceae) was collected from Ira village, situated in Bantwal taluk of Mangaluru, Karnataka, India. Fresh and disinfected plant material was gathered and transported to the laboratory in sterile zipper bags to preserve its integrity. The plant was identified by a qualified taxonomist using its distinctive features, including the flowers, pods, bark, and leaves [10]. In our previous study, fungal endophytes were isolated and identified as *N. clavispora* using morphological and molecular methods. In that study, the culture filtrate was extracted with ethyl acetate to obtain a crude extract for further investigation [11].

### Ultra pressure liquid chromatography: Mass spectrometry (UPLC-MS) analysis

A UPLC system, (Waters ACQUITY, USA) which featured a quaternary pump, an online degassing unit, an automated sampler, and a column compartment with precise temperature regulation was used for performing chromatographic analysis. In preparation for UPLC-MS analysis, the crude extract was diluted in methanol at a concentration of 1 mg/ml. ACQUITY UPLC® BEH C18 1.7μm column was used for the separation of bioactive components. The mobile phase consisted of solvent A (water containing 0.01% formic acid and 0.05% ammonia) and solvent B (acetonitrile with 0.1% formic acid), sourced from Supelco (Germany) and Biosolve BV (France). The elution gradient was carefully optimized based on these solvents. A constant flow rate of 0.35 mL/min was maintained throughout the run using gradient elution. The injection volume was set at 0.1 µL. The gradient profile applied was as follows: from 0 to 2 minutes, the ratio of solvents (A:B) was 95.0:5.0%; from 2 to 20 minutes, it changed to 5.0:95.0%; maintained at 5.0:95.0% from 20 to 30 minutes; and reverted to 95.0:5.0% between 30 and 40 minutes. Column temperature was held at 45°C. Mass spectrometric detection was performed using the UNIFI Scientific Information System with an electrospray ionization positive mode (ESI+). The collision energy was fixed at 6 eV, the cone voltage at 30 V, and the capillary voltage at 3.00 kV. The mass analysis range extended from 2 to 1200 Da. The ion source temperature was maintained at 100°C, whereas the desolvation temperature was set at 250°C. Desolvation and cone gas flow rates were controlled at 600 L/h and 50 L/h, respectively. Compound identification was achieved through accurate mass matching (±5 ppm), retention time alignment (±0.2 min), and UNIFI database comparison [12].

### Free radical scavenging activity by DPPH

Based on the method described by Blois (1958) [13], 1, 1-diphenyl-2-picrylhydrazyl (DPPH) assay was used to investigate the fungal extracts’ ability to scavenge free radicals. To test solutions (0–100 μg/ml), a freshly made DPPH (Himedia, India) solution (0.004% w/v) in 99% ethanol (Hayman,India) was added. The mixture was incubated at room temperature in the dark for 20 minutes. After this period, it was mixed thoroughly using a vortex mixer, and the absorbance at 517 nm was measured using a ultraviolet-visible absorption spectrometer (Thermo Scientific Genesys 180, USA). The blank was made of 99% ethanol. To have the same volume without any extract, a control sample was maintained. Ascorbic acid (Loba Chemie, India) was used as a reference standard. Each experiment was performed in triplicate. The following formula (1) was used to determine the percentage of the DPPH free radical that was scavenged:


$$\begin{array}{c} \text{DPPH radical scavenging activity }(\%)\;=\;({\text{A}}_{\text{control}}\text{-}{\text{A}}_{\text{test}})\;/\;{\text{A}}_{\text{control}}\;\times \text{ 100}. \end{array}$$ (1)

### The SOD protein's PDB structure selection and preparation

The 3D structure of SOD was obtained from protein data bank (https://www.rcsb.org/) with PDB ID: 5VF9 in pdb format was initially processed by eliminating all heteroatomic elements and subsequently examined using PYMOL visualization tool. After the removal of water molecules, polar hydrogen atoms were added to the purified protein model, and Kollman charges were assigned using AutoDock 4 (version 4.2.6). Each atom was assigned Kollmann partial charges of 9.0, and the finalized molecular structure was stored in the “PDBQT” format for forthcoming computational analyses [14].

### Ligand preparation

The 3D structures of the ligands ascorbic acid (AA) and cimicifoetiside B(CIM) were obtained from PubChem (https://pubchem.ncbi.nlm.nih.gov/) in SDF format. The ligands were rendered and analyzed in PYMOL, then exported as three-dimensional PDB files labeled Ascorbic_Acid.PDB and Cimicifoetiside_B.PDB. Using AutoDock 4 (version 4.2.6), polar hydrogen atoms were incorporated, and Gasteiger partial charges of −0.9987 for ascorbic acid and −1.0001 for cimicifoetiside B were assigned. Root torsions were defined, the total torsion count was evaluated, and a maximum permissible torsion limit was established for each ligand. Finally, each processed ligand was saved in the “PDBQT” file format [15].

### Molecular docking

Molecular docking studies of ascorbic acid and cimicifoetiside B with the SOD protein (PDB ID: 5VF9) were performed using AutoDock 4 (version 4.2.6). The protein active site was predicted using CASTp (http://sts.bioe.uic.edu/castp/) [16]. The binding site on the ligand was defined by establishing a grid box measuring 44 × 40 × 40 points along the x, y, and z axes, centered at coordinates x = 41.955, y = 45.209, and z = 91.415, with a grid spacing of 0.375 Å. All necessary docking parameters were included in the configuration file. The docking simulations were conducted using the Lamarckian Genetic Algorithm, with a population size of 150, a maximum of 2,500,000 energy evaluations, and a limit of 27,000 generations. The mutation and crossover probabilities were set to 0.02 and 0.8, respectively. A minimum of 100 independent runs of the genetic algorithm were executed. The docking results were stored in the parameter file ‘protein_ligand.dpf’. Following the completion of AutoGrid and AutoDock processes, a dlg file was generated containing the binding energies for the predicted protein-ligand complexes. [17]. The obtained complex file was saved in .pdb format for further analysis.

### Molecular dynamic (MD) simulation

Molecular dynamics simulations were carried out using GROMACS version 2023.2, with all required input files generated via the CHARMM-GUI web-based interface (https://www.charmm-gui.org/) [18]. This web-based tool provides an interactive interface for assembling molecular systems and calculating absolute binding free energies utilizing CHARMM [19]. All-atom molecular dynamics simulations were performed using the docked complexes of SOD bound to ascorbic acid (5VF9-AA) and cimicifoetiside B (5VF9-CIM).

The protein–ligand complex was embedded in a rectangular water box with a 10 Å buffer between the complex and the box edges. The system was parameterized using the CHARMM General Force Field (CGenFF) and solvated with the three-point TIP3P water model. To mimic physiological conditions, 0.15 M KCl was introduced, and a total of 16 potassium ions and 6 chloride ions were added to neutralize the system. These ions were arranged in a cubic crystal lattice configuration. The simulation box measured 54 Å along each edge (A, B, and C), with lattice angles (α, β, γ) set to 90°, implementing periodic boundary conditions in all three dimensions to simulate an infinite system. Electrostatic interactions were calculated using the particle-mesh Ewald (PME) method, with a Fourier grid spacing of 0.12 and a PME interpolation order of 4. A 1.2 nm cutoff was applied for van der Waals forces.

The simulation proceeded through equilibration and production phases. During equilibration, the Verlet cutoff scheme was applied; temperature was controlled using the Nose-Hoover thermostat, and pressure was regulated via the Parrinello-Rahman barostat. Both NVT (constant volume and temperature) and NPT (constant pressure and temperature) ensembles were used at 303.15 K. The GROMACS input files (version 3.7) are organized under the “GROMACS” folder in the “Charmm-gui.tgz” package. The production run lasted 100 nanoseconds, spanning 50 million steps. All simulations were conducted in triplicate, and the resulting data were analyzed and reported as mean ± standard deviation. Computational experiments were carried out on a custom-built workstation featuring an Intel Core i9-12900K CPU, Corsair H100 RGB cooling system, Gigabyte Z790UD AX DDR5 motherboard, 64 GB of Corsair DDR5 5200 MHz RAM (2x32 GB), a 1 TB Samsung 990 M.2 NVMe SSD, a 2 TB Western Digital SATA 7200 RPM hard drive, and an MSI RTX 4070i Gaming X Trio 12 GB GPU. [20].

### Assessment of binding free energy and decomposition

The binding free energies of the 5VF9-AA and 5VF9-CIM complexes were calculated using the Molecular Mechanics Poisson–Boltzmann Surface Area (MM-PBSA) method. A new trajectory was generated from the original file using the gmx-trjconv module, and frames were sampled at 10 ns intervals, yielding 100 frames from 1000 total frames. The Adaptive Poisson–Boltzmann Solver (APBS) was used for the electrostatic solvation calculations and gmx_MMPBSA version 1.6 was used to protein was cperform the molecular dynamics trajectories and perform the binding free energy analysis [21]. MM-PBSA calculations were performed for the 5VF9-AA and 5VF9-CIM complexes as well as the apo protein, to estimate the binding free energy. The binding free energy was decomposed into molecular mechanics and solvation energy contributions, including polar and nonpolar terms, according to Equation (2) and (3)


$$\begin{array}{c} {\Delta \text{G}}_{\text{Binding}}={\text{G}}_{\text{complex}}\;-\;({\text{G}}_{\text{protein}}\;+\;{\text{G}}_{\text{ ligand}}) \end{array}$$ (2)

$$\begin{array}{c} \Delta \text{G}={\Delta \text{E}}_{\text{MM}}+{\Delta \text{G}}_{\text{solvation}} \end{array}$$ (3)

The equation presented outlines different energy components involved in the interaction between a ligand and a protein. Here, ΔG_Binding_ denotes the free energy of binding, G_complex_ corresponds to the total energy of the ligand-protein complex, and G_protein_ along with G_ligand_ indicate the total free energies of the protein and ligand individually [22]. ΔE_MM_ refers to the average molecular mechanics energy calculated including electrostatic and van der Waals interactions, while G_solvation_ denotes the solvation free energy [23]. MM-PBSA calculations were performed for the 5VF9-AA and 5VF9-CIM complexes, as well as the apoprotein.

### Principal component analysis (PCA)

Principal Component Analysis (PCA) offers an understanding of the general motions and dynamic patterns exhibited by both the apoprotein and the protein–ligand complex throughout the simulations, linking these movements to their biological roles [24]. This method was applied to predict critical coordinated motions that take place during ligand binding. Initially, eigenvectors were calculated to represent the overall protein movements. The analysis focused on identifying the most interconnected motions within both the protein and its complex over the course of the simulations. After performing diagonalization of the covariance matrix of atomic fluctuations, the eigenvalues were derived. These principal components emphasize significant conformational changes distinguishing the protein from the complex and help characterize coordinated atomic movements. The principal components are obtained by diagonalizing the covariance matrix, denoted as C.


C = V^V T (4)

The eigenvalues occupy the diagonal positions of the matrix, whereas the matrix V holds the associated eigenvectors [25]. The eigenvector linked to the highest eigenvalue, which corresponds to the first principal component, captures the maximum variance within the dataset. PCA was performed for both the apoprotein and the protein–ligand complexes.

### Gibbs free energy

The free energy landscape (FEL), illustrating the most stable and lowest-energy conformations, was generated using the g_sham module. Variations in enthalpy (ΔH), standard free energy (ΔG), and entropy (ΔS) were determined based on Equation (5).


$$\begin{array}{c} \Delta \text{G}=\Delta \text{H T}\Delta \text{S} \end{array}$$ (5)

In this context, ΔH represents enthalpy, T corresponds to the temperature in Kelvin, ΔS signifies entropy, and ΔG denotes Gibbs free energy. The lowest Gibbs free energy values for the first two principal components (PC1 and PC2) indicate that the complex is thermodynamically stable [26]. The Gibbs free energy was calculated, and the resulting plots for the 5VF9-AA and 5VF9-CIM complexes were analyzed to assess the thermal stability of the protein. [27].

### Ethical statement and experimental animals

The study was approved by the Institutional Animal Ethics Committee (KSHEMA/IAEC-02/2023/06; Registration No. 115/PO/ReBi/99/CPCSEA), and all animal experiments adhered to ARRIVE guidelines. Healthy male Swiss albino mice (Mus musculus), aged between 6 and 8 weeks and weighing around 26 ± 2 grams, were procured from the Animal House of KS Hegde Medical Academy, Mangalore, Karnataka, India. The animals were maintained under controlled environmental conditions, with a temperature of 25 ± 2°C and a light-dark cycle of 14 hours light and 10 hours darkness. They received standard pelleted rodent diet and were provided with unrestricted access to water throughout the study. All measures were taken to minimize animal suffering. No mouse exhibited >20% body weight loss from baseline or impaired access to food/water. Health was monitored daily, animals meeting humane endpoint criteria were euthanized within 24 h by intraperitoneal injection of ketamine (100 mg/kg) and xylazine (10 mg/kg) in PBS (100 μl) [28]. No unplanned deaths occurred. All personnel completed mandatory training and held certification for animal research.

### Evaluation of acute toxicity

The acute oral toxicity study was performed according to OECD Guideline 425 (OECD, 2001). Male Swiss albino mice were divided into two groups (n = 3). Group I received 0.5% DMSO and served as the control, while Group II was orally administered *N. clavispora* extract (2000 mg/kg body weight). Animals were monitored for signs of toxicity and mortality for 14 days. As no significant toxic effects or mortality were observed, 100 mg/kg body weight was selected for subsequent *in vivo* studies [29].

### Groupings and irradiation parameter

Six mice in each group received a daily administration of Ascorbic acid and NC extract at a dose of 100 mg/kg body weight before being subjected to a sublethal gamma radiation exposure of 2 Gy. The study was designed with five distinct animal groups, described as follows.

- Group C – Control – untreated.
- Group DC – Drug control – animals are administered with 100 mg/kg bodyweight /day dose of NC extract for 5 days.
- Group RC – Radiation control – animals are administered with distilled water for 5 days, and on the 5th day, irradiated with 2 Gy gamma radiation.
- Group AA – Standard – animals are orally administered with 100 mg/kg bodyweight /day dose of Ascorbic acid (AA) for 5 days and on the 5th day, irradiated with 2 Gy gamma radiation.
- Group NC – Test Group – animals are orally administered with 100 mg/kg bodyweight /day dose of NC extract for 5 days and on the 5th day, irradiated with 2 Gy gamma radiation.

The Swiss albino mice were subjected to gamma irradiation at a dose of 2 Gy using the Low-Dose Irradiator 2000, developed by the Board of Radiation and Isotope Technology (BRIT) in India. The irradiation procedure was carried out at a rate of 3.58 Gy per minute at the Centre for Application of Radioisotopes and Radiation Technology (CARRT) facility, situated at Mangalore University, Karnataka. At the end of the experimental period (day 6), all animals were euthanized within 24 hours after irradiation. Prior to euthanasia, anesthesia was administered intraperitoneally using ketamine (100 mg/kg) in combination with xylazine (10 mg/kg). Whole blood was collected through cardiac puncture for Biochemical assessment.

#### Estimation of SOD activity by Nitroblue Tetrazolium Method.

The assay employs nitro blue tetrazolium chloride (NBT) as the substrate, which interacts with superoxide anions generated by riboflavin under light exposure, in the presence of methionine serving as an electron donor. This reaction produces a blue formazan complex. SOD present in the sample scavenges these superoxide radicals, leading to a decrease in formazan formation, which is reflected by a diminished intensity of the blue coloration. For assay preparation, a 10% liver homogenate is created by homogenizing 1 g of liver tissue in 10 mL of 0.4 M phosphate buffer at pH 7.0. The homogenate is then centrifuged at 10,000 rpm for 15 minutes, and the resulting supernatant is utilized for the assay. The subsequent reactions are established as described below.

- **Test:** 0.3 ml riboflavin, 2.5 ml methionine, 0.1 ml NBT, and 0.1 ml liver homogenate.
- **Control:** 0.3 ml riboflavin, 2.5 ml methionine, 0.1 ml 0.05M phosphate buffer, and 0.1 ml liver homogenate.
- **Standard:** 0.3 ml riboflavin, 2.5 ml methionine, 0.1 ml NBT, and 0.1 ml 0.05M phosphate buffer.
- **Blank:** 0.3 ml riboflavin, 2.5 ml methionine, and 0.2 ml 0.05M phosphate buffer.

Place the test, standard, and control mixtures in an aluminum foil-lined chamber with a 15W fluorescent light bulb and expose them to light for 10 minutes. After illumination, immediately measure the optical density at 560 nm. Use the provided formula to calculate the enzyme activity in the sample and express the results in units per milligram of protein (U/mg protein) for liver homogenate [30].


$$\begin{array}{c} \text{SOD activity}/\text{mg of protein }=\;\frac{\text{SOD activity }\times \;\text{5}}{\text{Total protein}} \end{array}$$ (6)

### Statistical analysis

Data are expressed as mean ± standard deviation (SD). Statistical comparisons among groups were performed using one-way ANOVA followed by Tukey’s post hoc multiple-comparison test in GraphPad Prism 8. Differences were considered statistically significant at p < 0.05.

## Results

### UPLC-MS analysis revealed multiple bioactive metabolites in the extract

**Fig 1:**
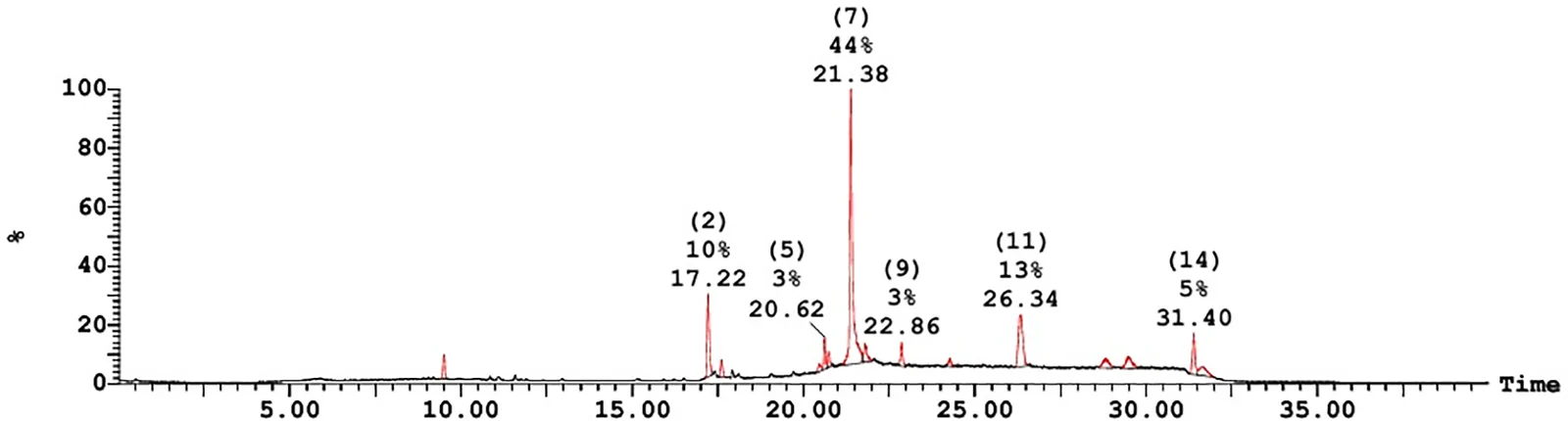
The representative chromatogram and base peak ion chromatograms of the *Neopestalotiopsis clavispora* endophytic fungal extract.

Secondary metabolites from *N. clavispora* extract were identified by UNIFI database matching using UPLC-TOF/MS [50]. The base peak ion chromatograms (Fig 1) serve as the analytical fingerprint for this species. The acquired mass spectrometry data were imported into the UNIFI information management platform. Within the UNIFI software, a theoretical compound database alongside a physical reference substance database specific to the *N. clavispora* extract was developed. Nine metabolites were identified using the UNIFI database (accurate mass ≤5 ppm, retention time ±0.2 min) as shown in Table 1.These included Chikusetsusaponin iva (Rt 17.22 min, m/z 398.2; 10%), Acetoxy gingerol (Rt 20.62 min, m/z 413.3; 3%), cimicifoetiside B (Rt 21.38 min, m/z 727.5; 44%), Tubulosine (Rt 22.86 min, m/z 493.4; 3%), Astin B (Rt 26.34 min, m/z 624.5; 13%), an unknown compound (Rt 31.40 min, m/z 223.9; 5%), Baicalein (Rt 9.50 min, m/z 287.06), Oroxylin A (Rt 17.61 min, m/z 285.10), Oroxylin B (Rt 20.46 min, m/z 594.2), and Chrysin-6-C-β-D-glucopyranosyl-8-C-α-L-arabinopyranoside (Rt 20.73 min, m/z 549.38). cimicifoetiside B was the predominant compound, comprising 44% of the total peak intensity.

**Table 1:** Bioactive compounds identified by database matching in *Neopestalotiopsis clavispora* endophytic fungal extract by UPLC-MS analysis.

Retention Time (min)	Mass [M + H] (m/z)	Compound Name	Literature reported biological activities
17.22	398.2	Chikusetsusaponin iva	Anti-inflamatory and cytotoxic. [31]
20.62	413.3	Acetoxy [10] gingerol	Antioxidant, Anticancer, Antibacterial, Anti-inflamatory. [32–34]
**21.38**	**727.5**	**Cimicifoetiside B**	**Anticancer, Antitumor,** **Cytotoxic. [**35–37**]**
22.86	493.4	Tubulosine	Anticancer and Antitumor. [38,39]
26.34	624.5	Astin B	Antitumor and Anticancer. [40]
31.40	223.9	Unknown	----
9.50	287.06	Baicalein	Antioxidant, Anticancer, Antitumor, Anti-inflamatory, neuroprotective. [41–43]
17.61	285.10	Oroxylin A	Anticancer, anti-angiogenic, anti-inflammation, anti-invasive and neuroprotective effects. [44–46]
20.46	594.2	Oroxylin B	Anticancer, Anti-inflamatory. [47,48]
20.73	549.38	chrysin-6-C-β-D-glucopyranosyl-8-C-α-L-arabinopyranoside	Anti-migraine. [49]

### DPPH free radical scavenging assay

**Fig 2:**
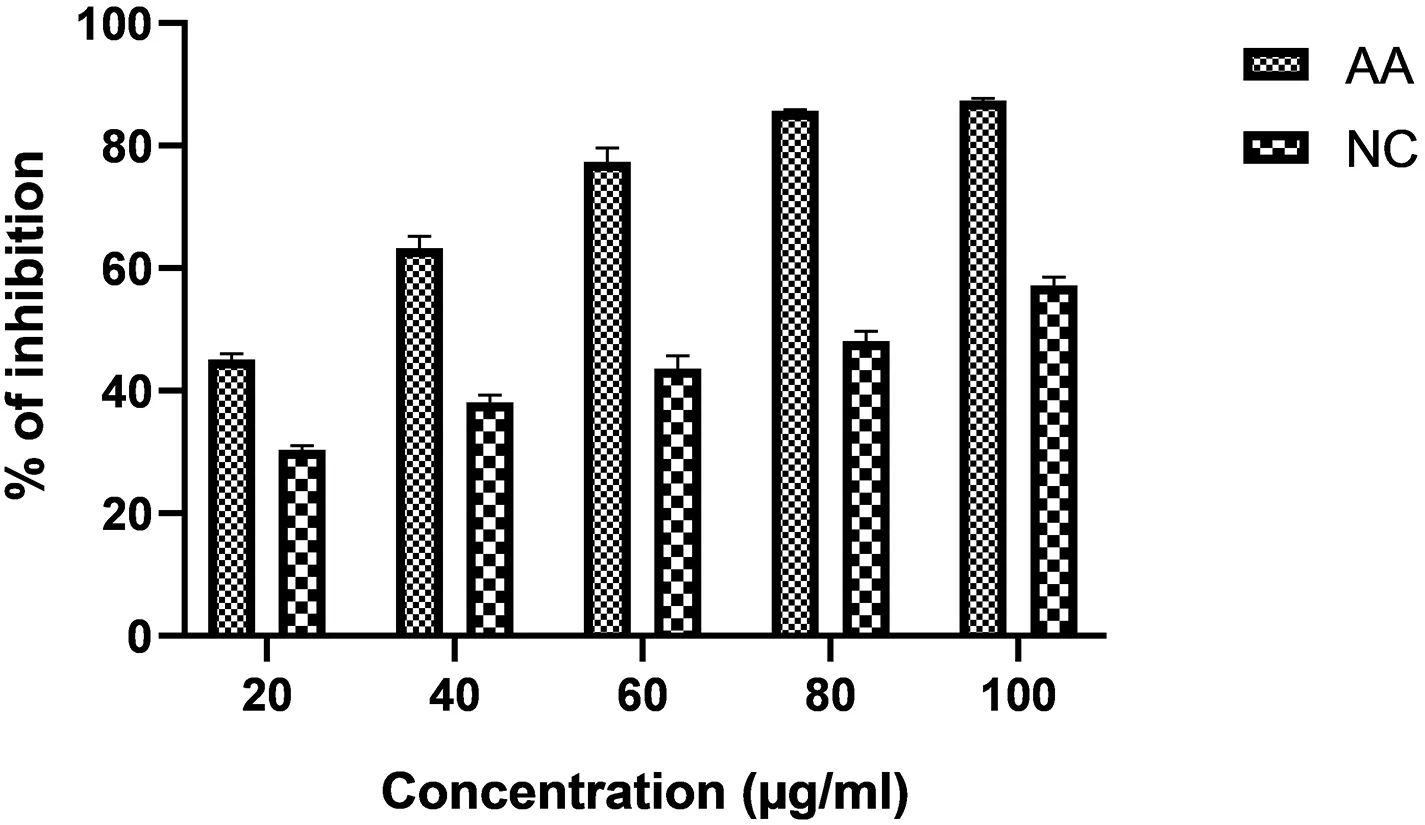
The free-radical scavenging activity of *Neopestalotiopsis clavispora* (NC) extract. The data are expressed as mean ± standard deviation.

The DPPH free radical scavenging activity of *N. clavispora* increased with concentration, reaching its peak at 100 µg/mL. At this concentration, *N. clavispora* exhibited a significant dose-dependent increase in antioxidant activity, with a scavenging percentage of 56.32 ± 2.39. Ascorbic acid was used as the reference antioxidant. In this assay, ascorbic acid showed 87.54 ± 0.30% radical scavenging activity at 100 μg/ml, which was the highest compared to *N.clavispora* extract. The IC₅₀ value of the DPPH assay for the *N. clavispora* extract was 82.86 μg/mL, whereas the reference antioxidant, ascorbic acid, exhibited an IC₅₀ value of 19.01 μg/mL (Fig 2). These findings indicate that the extract possesses antioxidant activity but with lower DPPH radical scavenging potency than ascorbic acid. Nevertheless, the DPPH results are consistent with the extract's previously reported antioxidant activities in ABTS, FRAP, superoxide anion scavenging, and total antioxidant capacity (TAC) assays [51], collectively demonstrating broad antioxidant potential across multiple radical scavenging mechanisms.

### Molecular docking

**Fig 3:**
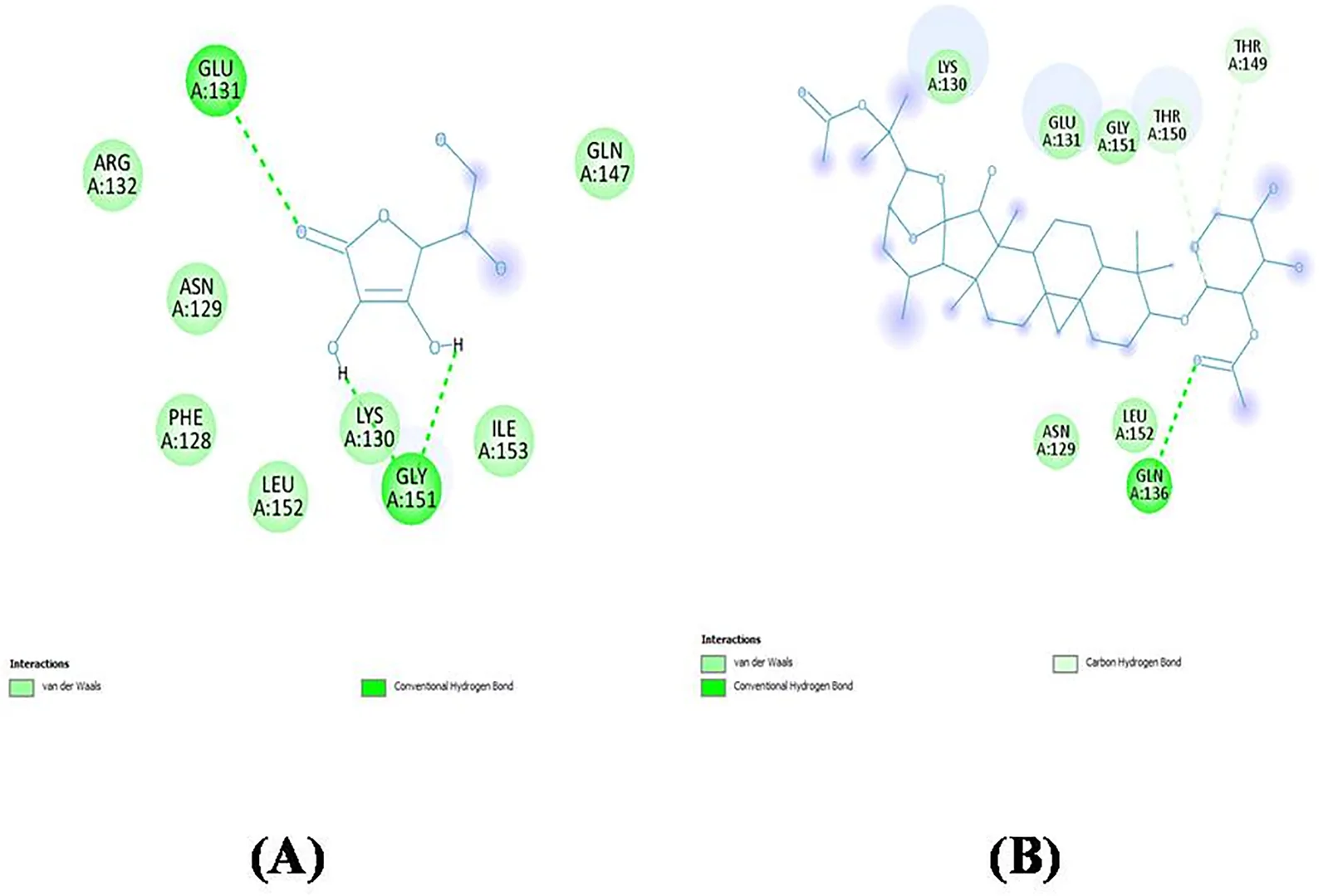
The molecular docking interactions of two ligand molecules with the SOD protein (PDB ID: 5VF9) were analyzed, and two-dimensional (2D) diagrams were generated to illustrate the ligand-protein interactions. Two-dimensional (2D) diagrams were generated to show the ligand-protein interactions of the 5VF9 target protein with (A) ascorbic acid and (B) cimicifoetiside B. In these diagrams, hydrogen bond interactions between the ligands and the protein residues are depicted using green dotted lines, pointing towards the electron donor atoms involved in the bonding.

The docking study was conducted to assess the binding affinities and molecular interactions of ascorbic acid and cimicifoetiside B with the 5VF9 protein. Both ascorbic acid and cimicifoetiside B exhibited notable interactions with the 5VF9 protein, with binding energies of −4.62 kcal/mol and −4.96 kcal/mol, respectively. While ascorbic acid formed three hydrogen bonds and cimicifoetiside B only one, cimicifoetiside B still showed comparable docking affinity. This suggests that factors beyond hydrogen bonding – such as hydrophobic interactions, molecular geometry, or overall binding affinity may significantly influence its binding effectiveness. Docking analysis (redocking-validated, RMSD < 2 Å are provided in the supplementary material as S1 Fig) reveals ascorbic acid and cimicifoetiside B binding to the CASTp-predicted SOD active site, comparable binding energies provides preliminary support for SOD as an interaction target (Fig 3). *In silico* molecular docking emerges as a pivotal technique in structural biology and drug development, facilitating the comprehension of molecular assembly [52]. The SOD binding site prediction and model evaluation offered valuable insights into the structural foundation of the protein-ligand interaction.

### MD simulation studies

Following molecular docking, the both complexes were selected for further MD simulations. MD simulations are useful for evaluating the structural integrity at the molecular level, interactions between the protein and ligand, changes in molecular shape or structure, behavior and movement of amino acid residues within the active region, and the precise spatial arrangement of the ligand within the protein’s binding pocket [53,54]. The triplicate results for RMSD, RMSF, hydrogen bonding, SASA, radius of gyration (Rg), and PCA analyses (S2-S7 Figs) and MM-PBSA results (S1- S2 Tables in S1 File) are presented as supplementary information.

### RMSD

**Fig 4:**
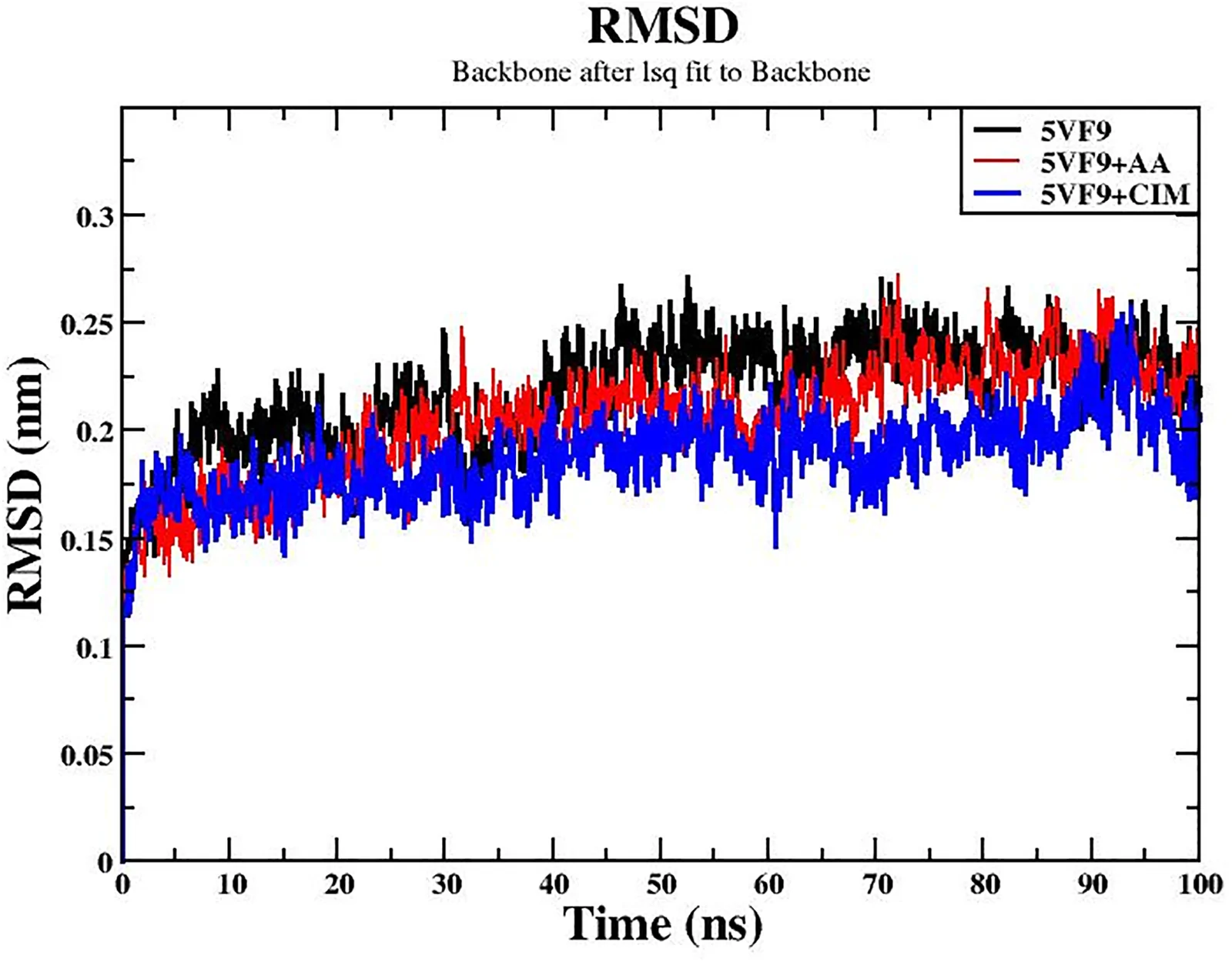
The results from the 100-nanosecond molecular dynamics simulations of the native 5VF9 protein and its complexes with 5VF9-AA and 5VF9-CIM are illustrated. During the simulations, the root mean square deviation (RMSD) of the alpha carbon atoms was monitored for both the unbound protein and the protein–ligand complexes. Time is plotted along the X-axis in nanoseconds, while the Y-axis displays RMSD values measured in nanometers. The RMSD curve for the native 5VF9 protein is shown in black, the 5VF9-AA complex in red, and the 5VF9-CIM complex in blue.

The average RMSD was calculated to assess deviations in the protein-ligand complexes over the 100 ns binding period, reflecting the overall stability of both the apo and complex forms. The apo protein exhibited an average RMSD of 0.21 ± 0.02 nm. For the 5VF9 complexed with ascorbic acid, the RMSD was 0.20 ± 0.02 nm, while for the complex with cimicifoetiside B, it was 0.18 ± 0.02 nm, (Fig 4.) Throughout the simulation, it was observed that the stability of the apo protein and the complexes with ascorbic acid and cimicifoetiside B showed minimal deviation, indicating that the binding of both ligands did not significantly alter the protein's stability. This suggests that the complexes remained as stable as the apo protein throughout the simulation. Lower RMSD values indicate stronger and more stable interactions between the protein and ligand, whereas higher RMSD values indicate reduced complex stability.

### RMSF

**Fig 5:**
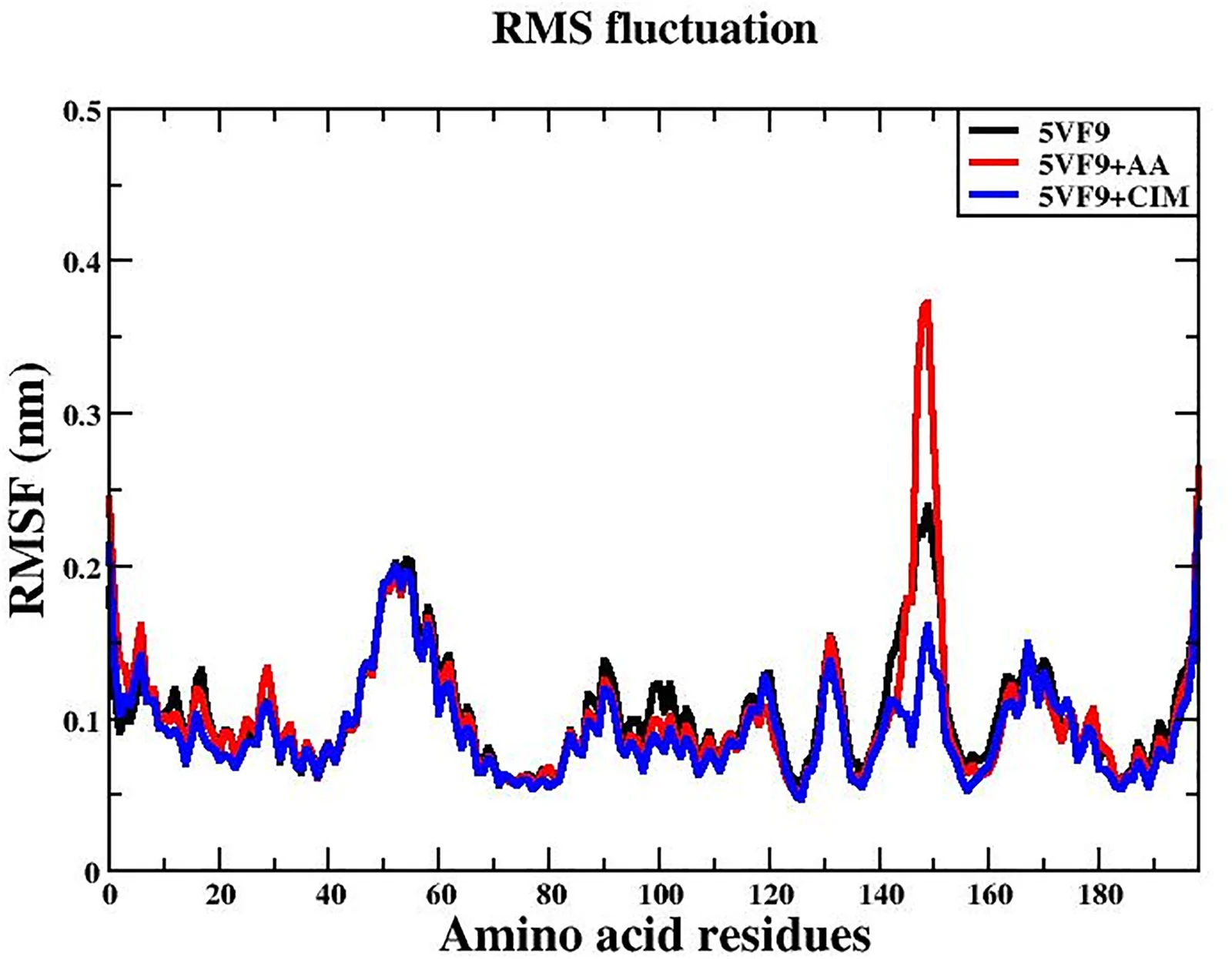
The 100 ns MD simulation results for the native 5VF9 protein and its complexes with ascorbic acid and cimicifoetiside B are shown. The Root Mean Square Fluctuation (RMSF) of the alpha carbon atoms in both the native protein and the protein–ligand complexes throughout the simulation are presented. The X-axis corresponds to the total atoms within the protein structure, and the Y-axis reflects the RMSF measurements in nanometers. The RMSF values for the native 5VF9 protein, the 5VF9-AA and the 5VF9-CIM complex are indicated in black, red, and blue, respectively.

Root mean square fluctuation (RMSF) was used to assess local conformational variations within the protein backbone. Analysis of the RMSF plot allows identification of the flexible regions within the protein structure. Typically, regions such as loops, turns, and coils exhibit higher RMSF values due to their inherent flexibility, while more rigid secondary structural elements like α-helices and β-sheets demonstrate lower fluctuations. To investigate the structural changes induced by ligand binding, RMSF profiles were generated for the native 5VF9 protein and its complexes with 5VF9-AA and 5VF9-CIM, as illustrated in Fig 5. The average RMSF values obtained were 0.10 ± 0.03 nm for 5VF9, 0.088 nm for 5VF9-AA, and 0.10 ± 0.03 nm for 5VF9-CIM. Overall, the fluctuation patterns observed in the apo form and both complexes were comparable.

### Hydrogen bond

**Fig 6:**
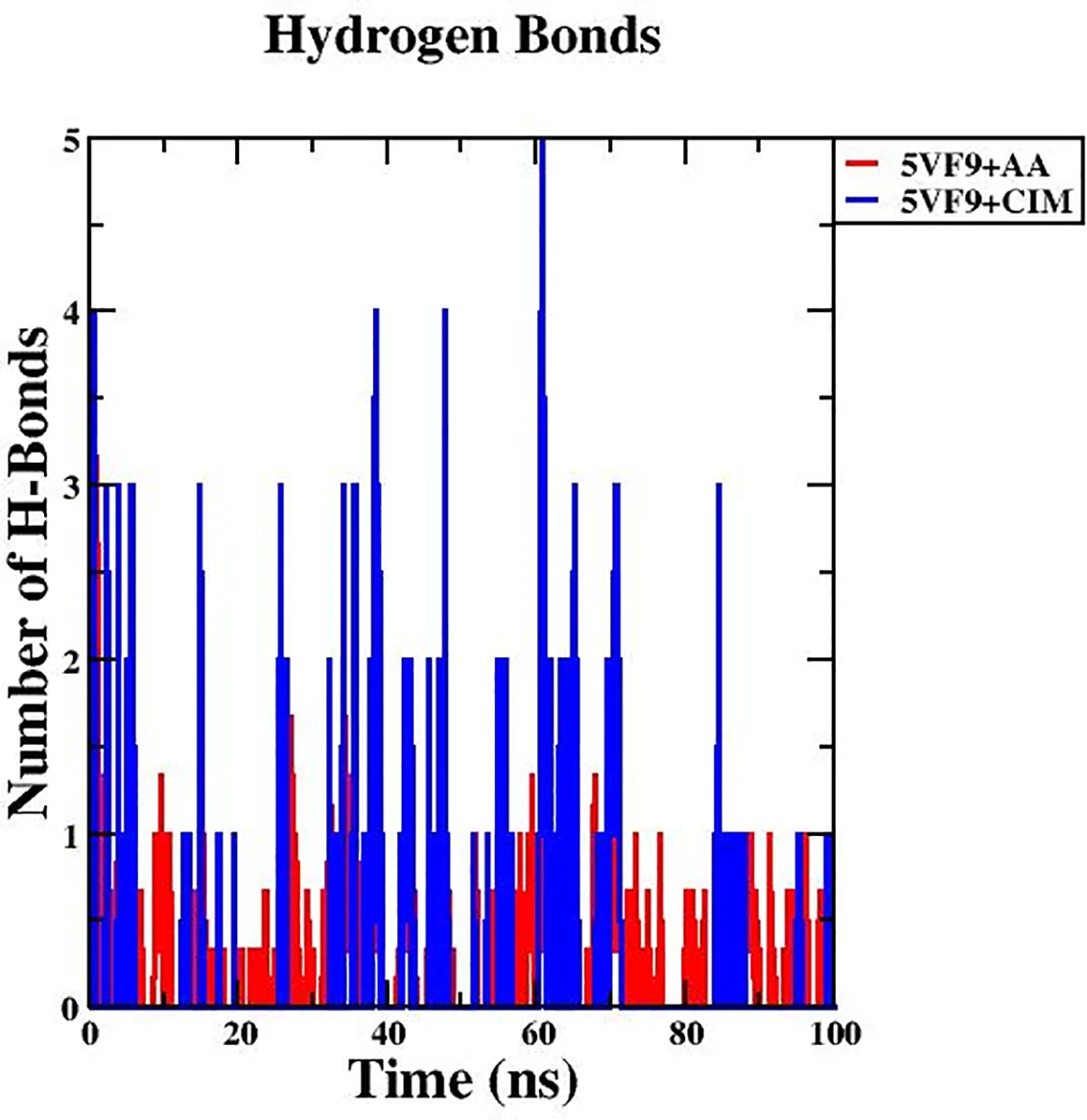
The 100 ns molecular dynamics simulation data for the native 5VF9 protein and its complexes with ascorbic acid and cimicifoetiside B are presented. An analysis of intermolecular hydrogen bonding over the simulation time frame is provided, with the X-axis representing the duration measured in nanoseconds and the Y-axis indicating the quantity of hydrogen bonds. The hydrogen bond interactions for the apoprotein 5VF9 are represented in black, while those for the 5VF9-AA complex are highlighted in red, and the 5VF9-CIM complex interactions are depicted in blue.

Hydrogen bonds are essential for maintaining the stability of the protein-ligand complexes. Fig 6 displays the count of hydrogen bonds formed within the 5VF9-AA and 5VF9-CIM complexes throughout the final 100 ns of the simulation. Hydrogen bonds were assessed using a cutoff distance of 3.5 Å and an angular threshold of 30° [55] to detect polar interactions within the 5VF9-AA and 5VF9-CIM complexes. The initial quantification of hydrogen bonds between the ligand and the protein showed dynamic fluctuations throughout the simulation. The hydrogen bonding profiles for the 5VF9-AA and 5VF9-CIM complexes, depicted in Fig 6, reveal consistent interactions. Throughout the 100 ns simulation, the 5VF9–CIM complex exhibited a greater number of hydrogen bonds than the 5VF9–AA complex. These observations confirm the formation of stable complexes, with hydrogen bonds facilitating ligand binding to the native 5VF9 protein.

### Solvent Accessible Surface Area (SASA)

**Fig 7:**
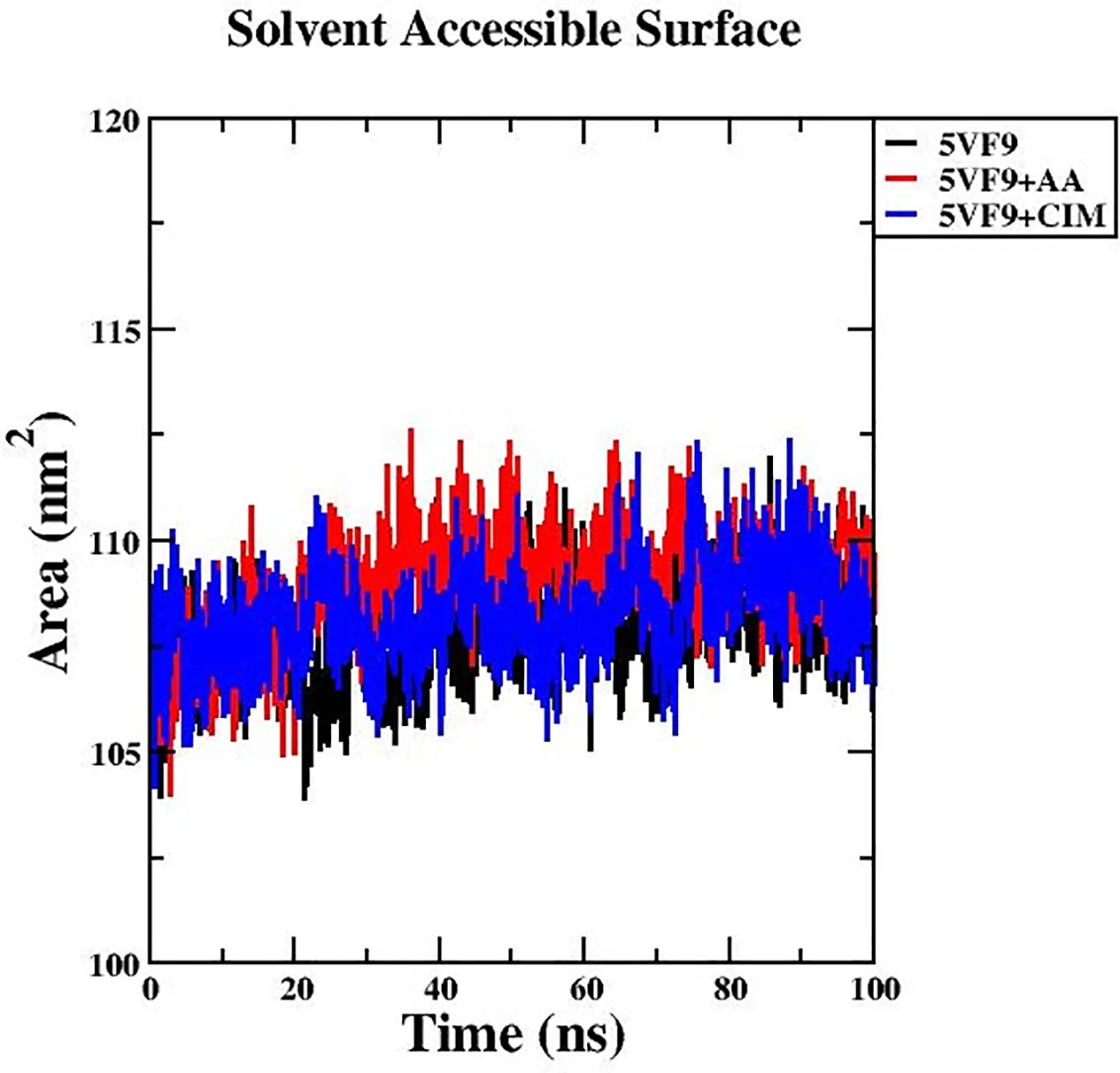
The 100 ns molecular dynamics simulation data for the native SOD protein and its complexes with ascorbic acid and cimicifoetiside B are presented. The solvent-accessible surface area, which reflects the portion of the protein surface in contact with the surrounding solvent, is plotted over time. The X-axis corresponds to the duration expressed in nanoseconds., while the Y-axis denotes the surface area in nm². The SASA of the native 5VF9 apoprotein is illustrated in black, the 5VF9-AA complex in red, and the 5VF9-CIM complex in blue, highlighting the respective interactions.

SASA reflects the degree of protein exposure to solvent, providing insights into protein unfolding during MD simulations. SASA reflects the solvent exposure of the protein and provides insight into conformational stability by indicating the extent of hydrophobic interactions with surrounding solvent molecules. [56]. The 5VF9-AA and 5VF9-CIM complexes exhibited relatively stable SASA profiles, with values of 108.96 ± 1.35 nm² and 108.27 ± 1.23 nm², respectively (Fig 7). These values show little variation compared to the native protein's SASA of 107.73. ± 1.18 nm², further reinforcing the consistent conformational behavior of the ligand-associated structures.

### Radius of Gyration (Rg)

**Fig 8:**
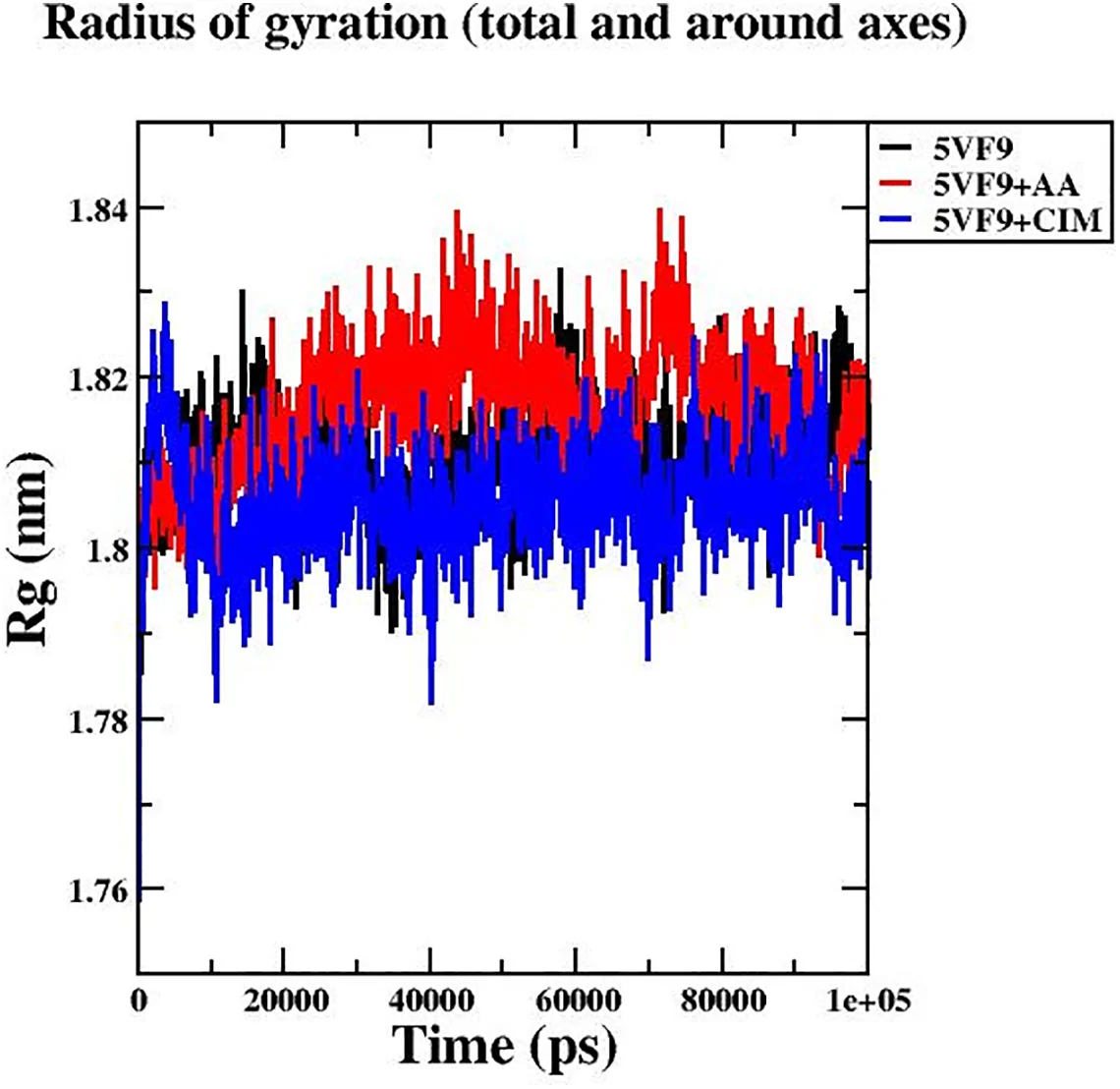
The 100 ns molecular dynamics simulation data for the native 5VF9 protein and its complexes with ascorbic acid and cimicifoetiside B are presented. The analysis utilizes the Radius of Gyration of the protein and its complexes to assess the impact of ascorbic acid and cimicifoetiside B on the 5VF9 target. The X-axis denotes the timescale in picoseconds, while the Y-axis represents the area in nanometers. The Radius of Gyration of the apoprotein is depicted in black, the 5VF9-AA complex in red, and the 5VF9-CIM complex in blue, thereby highlighting the distinct interactions observed.

The radius of gyration is a valuable parameter for evaluating the structural integrity and compactness of the systems under study. It is defined as the mass-weighted root mean square distance of atoms from the system's center of mass. The Rg values reflect the degree of compactness of the protein structure both before and after ligand binding. The average Rg values obtained were 1.55 ± 0.03, 1.56 ± 0.05 and 1.55 ± 0.03 nm for 5VF9, 5VF9-AA, and 5VF9-CIM, respectively (Fig 8). The Rg analysis revealed that the compactness of the 5VF9-CIM complex is similar to that of the 5VF9-AA complex.

### MM-PBSA

The MM-PBSA calculations were performed to conduct a comprehensive analysis of the MD data for the 5VF9-AA and 5VF9-CIM complex. Table 2 summarizes the interaction energies obtained from the MD trajectories, including binding, electrostatic, polar solvation, and van der Waals energies. According to the MM-PBSA results, the 5VF9–AA complex exhibited a binding energy of −1.24 ± 0.13 kcal/mol, whereas the 5VF9–CIM complex showed a significantly higher binding energy of −12.36 ± 2.32 kcal/mol. These findings suggest that cimicifoetiside B possesses a stronger binding affinity toward 5VF9 compared to AA.

**Table 2:** Binding energy together with other energy contributions factors for the 5VF9-AA and 5VF9-CIM complexes were determined using the MM-PBSA method.

Type of energy	Ascorbic acid Energy contributed (kcal/mol)	Cimicifoetiside B Energy contributed (kcal/mol)
van der Waal energy	−2.18 ± 0.25	−19.70 ± 4.72
Electrostatic energy	−4.54 ± 0.61	−8.98 ± 4.47
Polar solvation energy	5.48 ± 0.87	16.3 ± 3.21
Binding energy	−1.24 ± 0.13	−12.36 ± 2.32

### PCA

**Fig 9:**
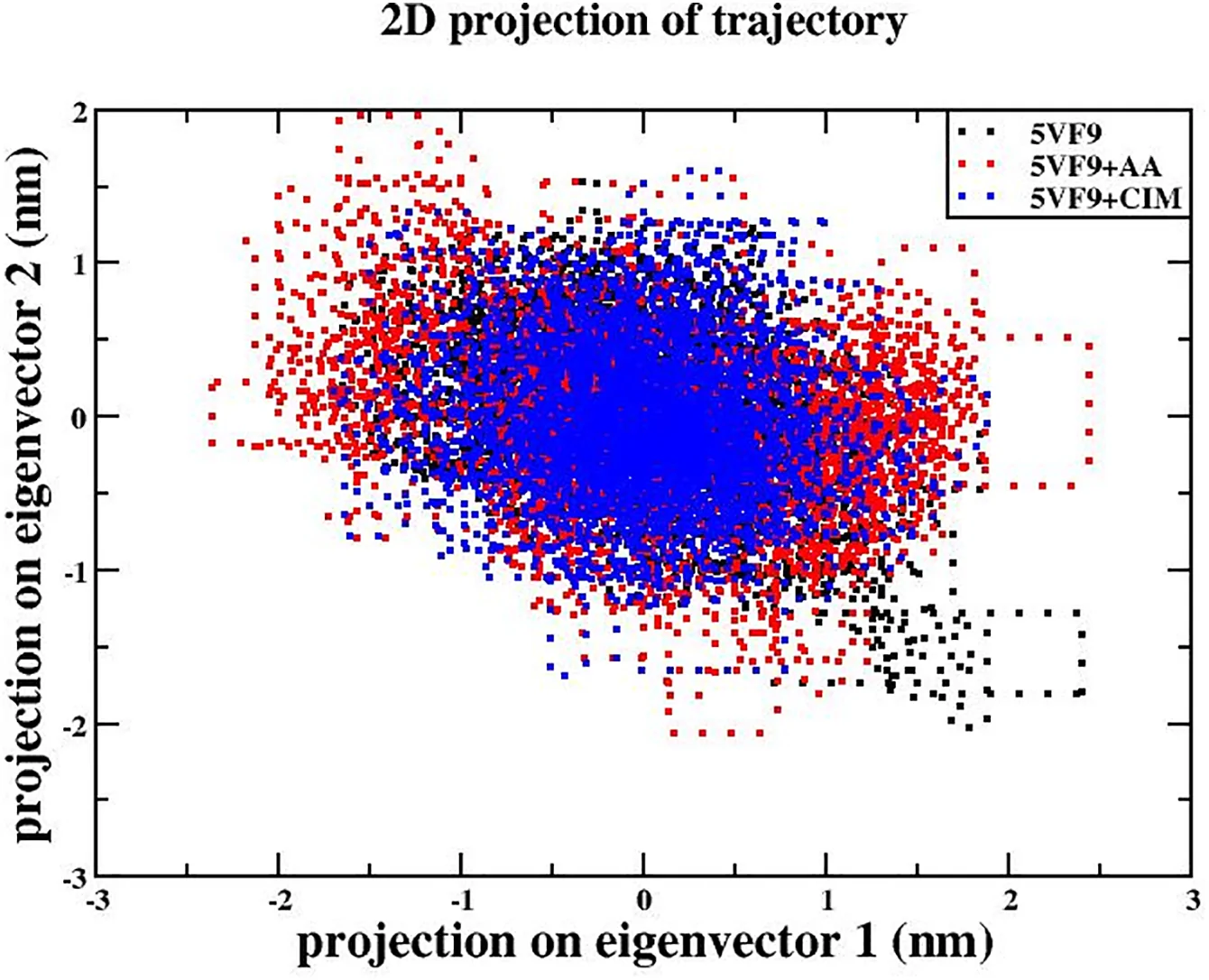
A 2D representation of the first two principal components (PC1 and PC2) for 5VF9 and its complexes with ascorbic acid and Cimicifoetiside B is depicted.

**Fig 10:**
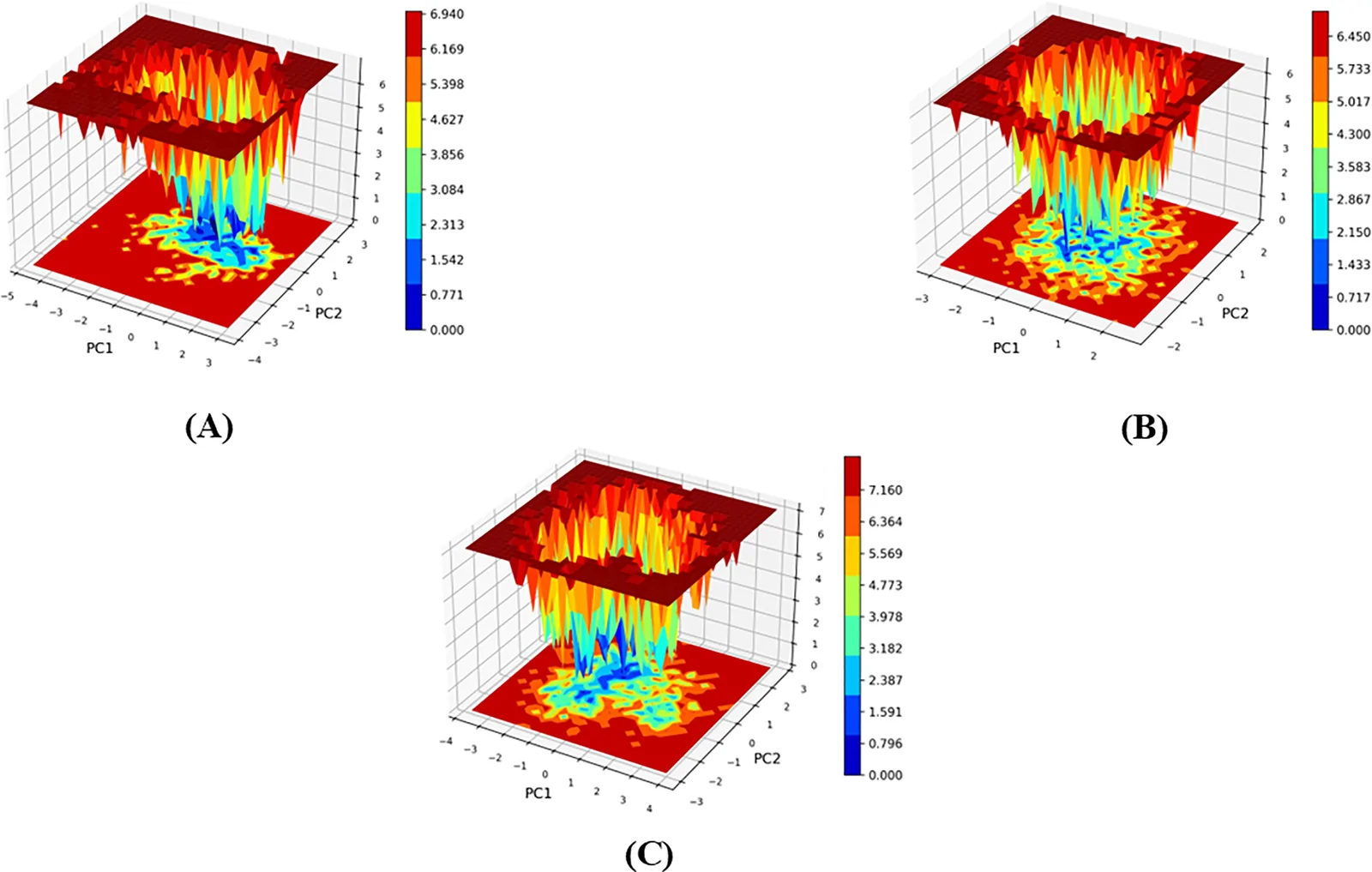
The free energy landscape (FEL) of the protein-ligand complexes: (A) 5VF9, (B) 5VF9 –AA and (C) 5VF9 -CIM. The color bar indicates the free energy values in kcal mol ^-1^.

PCA was carried out to capture the dominant collective motions associated with ligand binding, providing insights into structural alterations that govern ligand–protein interactions. Furthermore, PCA highlights the major dynamic modes that contribute to the functional behavior of proteins. A two-dimensional (2D) projection of the first two principal components (PC1 and PC2), corresponding to the eigenvalues, was generated for the 5VF9–AA and 5VF9–CIM complexes (Fig 10). In this plot, each data point denotes a distinct conformation of 5VF9 in its apo form and ligand-bound states across the 100 ns simulation trajectory. In the 5VF9, 5VF9–AA, and 5VF9–CIM systems, the vectors are mainly clustered within the central region ranging from −2–2 nm on both axes, indicating that the principal component motions in the ascorbic acid and cimicifetiside B complexes remain well-centered and less dispersed. This suggests that both ligand-bound forms exhibit stability comparable to the apo protein. As illustrated in Fig 9, ligand binding promotes the formation of more compact and stable clusters within the protein structure.

### Gibbs free energy

Over the MD simulation, the energy state distribution of the protein and complexes was analyzed using GROMACS's essential dynamic analysis. Various conformational forms exhibiting lower Gibbs energies are indicated by plots with a deeper blue color. The apoprotein exhibits an energy range of 0 to 6.94 kJ/mol, whereas the 5VF9-AA complex shows an energy range of 0 to 6.45 kJ/mol, and the 5VF9-CIM complex has an energy range of 0 to 7.16 kJ/mol. This indicates that the 5VF9-CIM and 5VF9-AA complex showed a similar transition pattern along with comparable Gibbs free energy and thermodynamic stability (Fig 10).

Binding free energy is the sum of all non-bonded interactions. The MD simulation results for RMSD, RMSF, H-bond, SASA, and Rg indicated that both AA and CIM are suitable, while PCA, MM-PBSA, and Gibbs free energy analyses showed optimal outcomes for both complexes. These results are consistent with our experimental observations, indicating that *in silico* docking predicts CIM to have a favorable binding affinity toward SOD, which may partly explain the observed effects of the extract.

### Estimation of SOD activity

**Fig 11:**
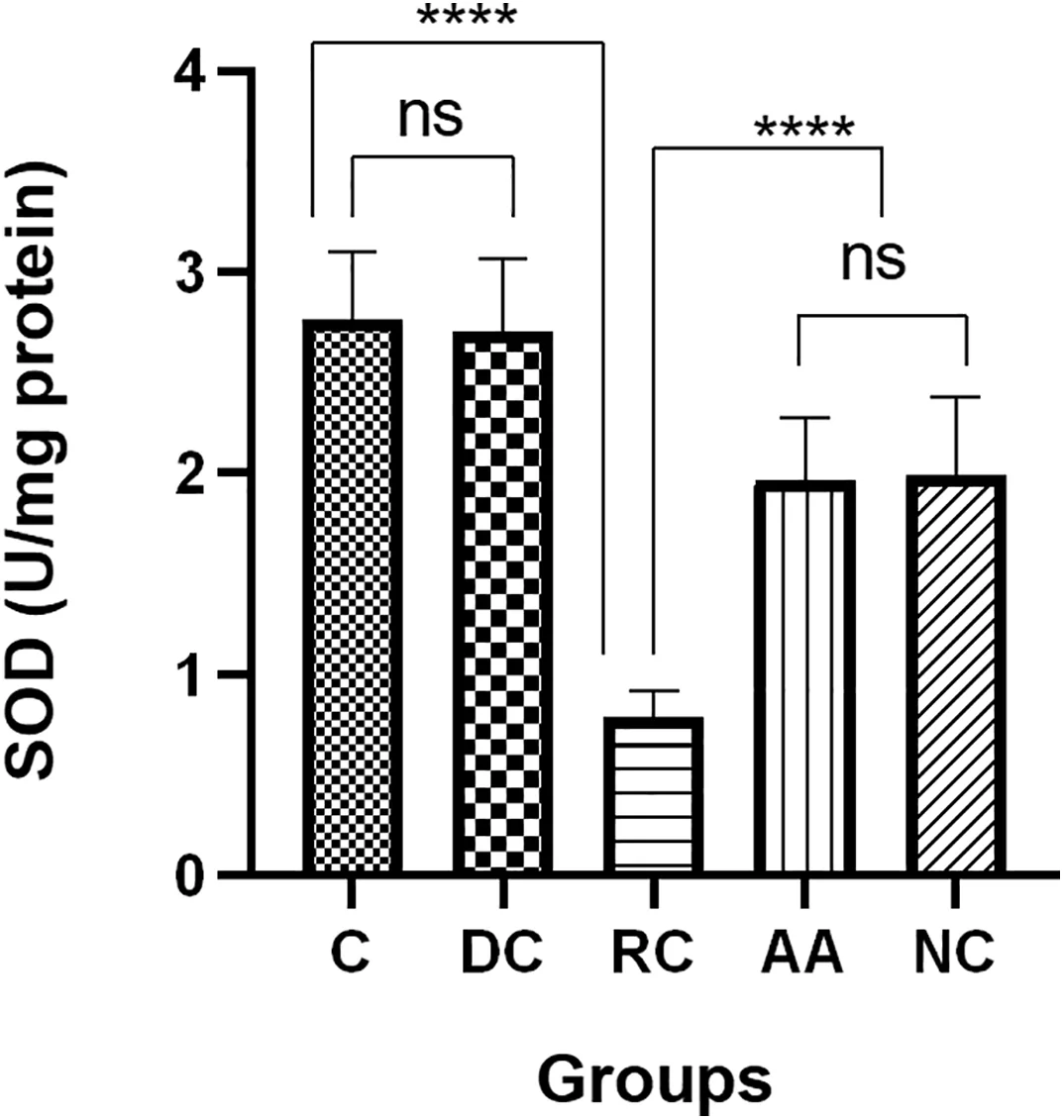
The activity of superoxide dismutase (SOD) in Swiss albino mice was evaluated under different experimental conditions. (C) Control, (DC) Drug control, (RC) Radiation control, (AA) Ascorbic acid (100 mg), and (NC) *N. clavispora* extract (100 mg). In the radiation control group, SOD activity exhibited a marked reduction (0.78 ±  0.13 U/mg protein) when compared with the normal control group (2.76  ±  0.33 U/mg protein; p < 0.0001). Administration of *N. clavispora* extract significantly increased SOD activity compared with the radiation control group (Tukey’s post hoc test, p < 0.0001), suggesting a protective antioxidant role. Meanwhile, no statistically significant variation was observed in the drug control group relative to the untreated control.

SOD is a crucial enzyme that helps mitigate oxidative stress by scavenging a wide range of free radicals. Significant changes in SOD activity were noted in the irradiated groups. The lowest SOD activity was recorded in the RC (Radiation control) group, with a value of 0.78 ±  0.13 U/mg protein. In contrast, an increase in SOD activity was observed in the AA (Ascorbic acid) group (1.96  ±  0.30 U/mg protein) and the NC group (1.98  ±  0.38 U/mg protein). However, the C (control) group exhibited a much higher SOD activity at 2.76  ±  0.33 U/mg protein, and similarly, the DC (Drug Control) group had a SOD activity of 2.71  ±  0.35 U/mg protein (Fig 11).

## Discussion

The ﬁrst in vivo studies on protection by antioxidants against ionizing radiation were performed a half century ago. Patt et al. reported in 1949 that the thiol amino acid, cysteine, protected rats from a lethal dose of X-rays. Discussions soon began concerning the actions of ionizing radiation on cells and how radioprotective chem-icals might help elucidate the mechanisms of interaction of radiation and molecules of biological importance. Gerschman and her co-workers hypothesized that both radiation injury and oxygen poisoning occur through the formation of reactive oxygen species. They demonstrated that antioxidants such as cysteine, glutathione, The present study examined the effectiveness of antioxidants in protecting tissues from radiation damage. Since radiation plays a crucial role in disease diagnosis and treatment in healthcare, minimizing its biological effects is essential [57]. Excessive production of ROS has been linked to the onset of several chronic and degenerative diseases, including cancer, respiratory disorders, neurodegenerative conditions, and digestive diseases. Under normal physiological conditions, ROS levels are tightly regulated by antioxidants, which can be produced internally or obtained from external sources [58]. The identification of the *Oroxylum indicum* plant was carried out by a taxonomist, and the plant specimen was documented for future reference. This study represents the first instance of utilizing UPLC-TOF/MS in conjunction with the UNIFI platform to analyze the chemical constituents of *N. clavispora* extract. The established method proved effective in identifying a substantial number of bioactive compounds. Earlier studies indicate that cimicifoetiside B exhibits cytotoxic activity against certain cancer cell lines, implying potential antiproliferative effects. Similar to other triterpene glycosides derived from *Cimicifuga* species, it may have the capacity to interact with estrogen receptors; however, the available data on this interaction remains limited. Currently, there is no detailed information on its role in antioxidant pathways or the mechanisms by which it may exert antioxidant effects. Additional research is needed to assess its possible antioxidant properties and to elucidate the mechanisms involved [35–37].

To investigate the interaction and possible mode of action of cimicifoetiside B with SOD, an antioxidant enzyme was chosen and analysed using computational approaches. Molecular docking studies are a valuable method for predicting the interaction between small molecules and target proteins. In this research, we conducted docking simulations using selected metabolites from *N. clavispora* with well-known antioxidant-related enzymes, SOD. Our findings revealed that the metabolite cimicifoetiside B exhibited favorable binding affinity toward the SOD enzyme. It should be noted that the antioxidant activity observed in this study was evaluated using the crude *N. clavispora* extract rather than purified cimicifoetiside B. Therefore, the observed antioxidant effects are likely the result of one or more bioactive metabolites present in the extract and cannot be attributed solely to cimicifoetiside B. Nevertheless, UPLC-MS analysis identified cimicifoetiside B as the predominant metabolite, and molecular docking together with molecular dynamics simulations demonstrated its favorable interaction with superoxide dismutase (SOD), suggesting that it may be one of the key contributors to the antioxidant activity. However, further bioactivity-guided fractionation, isolation of individual compounds, and experimental validation are required to confirm the specific metabolites responsible for the observed biological effects. The molecular dynamics simulations offered critical insights into the stability and dynamic behavior of the protein-ligand complexes identified during docking studies. Over the course of the simulation period (100 ns), the RMSD values indicated that the complexes remained stable, with minimal fluctuations, suggesting a strong and sustained interaction between the ligand(s) and the active site of the target protein. RMSF analysis further revealed that key binding site residues exhibited limited flexibility, supporting the notion of a stable binding conformation. Additionally, analysis of the radius of gyration and hydrogen bonding patterns reinforced the structural compactness and consistent interactions throughout the simulation. Notably, the 5VF9–CIM complex exhibited a higher binding free energy (−12.36 ± 2.32 kcal/mol) than the 5VF9–AA complex (−1.24 ± 0.13 kcal/mol), while the remaining interaction energy components showed similar trends for both ligands. Although cimicifoetiside B formed only a single hydrogen bond with SOD, the complex-maintained stability throughout the simulation that was similar to that of ascorbic acid. Overall, the in-silico findings suggest that cimicifoetiside B displays stable interactions and binding characteristics similar to ascorbic acid. These results support the docking observations and provide additional insight into the potential interactions of the phytochemicals identified through UPLC-MS with their molecular targets.

SOD serves as a crucial defense mechanism against oxidative stress. They neutralize superoxide radicals highly reactive molecules capable of damaging cellular structures like lipids, proteins, and DNA by converting them into hydrogen peroxide (H_2_O_2_) and oxygen (O_2_), thus reducing their harmful effects [59]. Since mitochondria are major producers of ROS due to their involvement in energy production, SODs play a vital protective role. Specifically, manganese superoxide dismutase (MnSOD), located in the mitochondrial matrix, safeguards mitochondrial DNA and proteins from oxidative injury, thereby supporting mitochondrial integrity and energy metabolism [60]. Various isoforms of SOD, including EcSOD (Extracellular Superoxide Dismutase), CuSOD (Copper Superoxide Dismutase) and MnSOD, have been extensively researched for their potential antitumor effects and their role in maintaining redox balance [61,62]. Post-radiation, the level of SOD activity can determine the extent of damage caused by radiation. The present study demonstrated a notable difference in SOD activity between animals treated with the *N. clavispora* extract and untreated animals. Specifically, the radiation control group showed the lowest SOD activity, while the animals that received *N.clavispora* extract treatment exhibited the highest SOD activity. The *in vivo* evaluation of SOD activity in liver tissues of irradiated mice provided valuable insights into the antioxidant potential of the *N. clavispora* extract. Radiation exposure is well-documented to induce oxidative stress by generating ROS, which disrupt cellular redox balance and damage macromolecules such as lipids, proteins, and DNA. In this study, a significant decrease in SOD activity was noted in the irradiated control group compared to the non-irradiated normal group and treated group, confirming the oxidative stress burden induced by radiation. Treatment with the *N. clavispora* extract preserved SOD activity, indicating a protective effect against oxidative stress. The results also suggest that constituents of the *N. clavispora* extract may interact with SOD in a way that supports the preservation of its functional stability under oxidative stress conditions. This improvement suggests that one or more phytochemicals identified by UPLC-MS, including flavonoids, glycosides, and phenolic compounds, may contribute individually or synergistically to the observed antioxidant activity through direct free radical scavenging or by enhancing endogenous antioxidant defense systems. The maintained SOD activity observed in the treated groups highlights the extract’s capacity to neutralize superoxide anions, thereby reducing oxidative damage in the liver and helping to maintain tissue integrity. Although the crude *N. clavispora* extract exhibited antioxidant activity, specific mechanisms (e.g., radical scavenging, metal ion chelation) were not investigated, limiting insights into its mode of action. Additionally, the present study primarily focused on SOD activity as a representative antioxidant marker, while other oxidative stress biomarkers such as CAT, GSH, and MDA were not evaluated and should be investigated in future studies for a more comprehensive understanding of the antioxidant mechanism. Key pharmacological aspects such as bioavailability, toxicity, and therapeutic potential of the dominant metabolite cimicifoetiside B remain unassessed. Future studies should prioritize compound isolation, mechanistic assays, and ADME/toxicity profiling to establish the extract's clinical translatability.

## Conclusion

The combined *in vitro*, *in silico*, and *in vivo* findings demonstrate that the crude *N. clavispora* extract possesses antioxidant-associated activity under the experimental conditions of this study. UPLC-MS analysis identified cimicifoetiside B as the predominant metabolite, and molecular docking together with molecular dynamics simulations suggested that this compound may interact with SOD. However, because the biological experiments were performed using the crude extract rather than purified cimicifoetiside B, the observed antioxidant activity cannot be attributed solely to this compound. It is likely that multiple metabolites present in the extract contribute individually or synergistically to the observed biological effects. Further bioactivity-guided fractionation, isolation of individual compounds, and experimental validation are required to identify the active constituents and clarify their mechanisms of action.

## Supporting information

S1 Fig Molecular docking interaction analysis for (A) Apo form of the protein, (B) Ascorbic acid-bound complex, and (C) CIM-bound complex prior to duplicate 100 ns MD simulations. (PDF)

S2 Fig Backbone RMSD analysis from triplicate 100 ns MD simulations for (A) Apo form of the protein, (B) Ascorbic acid-bound complex, and (C) CIM-bound complex. (PDF)

S3 Fig Residue-wise RMSF analysis from triplicate 100 ns MD simulations for (A) Apo form of the protein, (B) Ascorbic acid-bound complex, and (C) CIM-bound complex. (PDF)

S4 Fig Hydrogen bond analysis from triplicate 100 ns MD simulations for (A) Apo form of the protein, (B) Ascorbic acid-bound complex, and (C) CIM-bound complex. (PDF)

S5 Fig Solvent accessible surface area (SASA) analysis from triplicate 100 ns MD simulations for (A) Apo form of the protein, (B) Ascorbic acid-bound complex, and (C) CIM-bound complex. (PDF)

S6 Fig Radius of gyration (Rg) analysis from triplicate 100 ns MD simulations for (A) Apo form of the protein, (B) Ascorbic acid-bound complex, and (C) CIM-bound complex. (PDF)

S7 Fig Principal component analysis (PCA) from triplicate 100 ns MD simulations for (A) Apo form of the protein, (B) Ascorbic acid-bound complex, and (C) CIM-bound complex. (PDF)

S1 File Supplementary Tables S1 and S2. MM-PBSA-derived binding free energies along with individual energy contribution parameters for the 5VF9–AA and 5VF9–CIM complexes obtained from triplicate molecular dynamics simulations. (PDF)
