# Supplementary material for: UPLC-MS metabolite profiling and antioxidant efficacy of Neopestalotiopsisclavispora isolated from Oroxylum indicum (L.) Kurz Stem Bark
Source: PLoS One. 2026 Sep 9;21(9):e0357595. doi: 10.1371/journal.pone.0357595 (PMC13557386; doi:10.1371/journal.pone.0357595)
Supplement: Supplementary file 5 [file pone.0357595.s005.pdf]

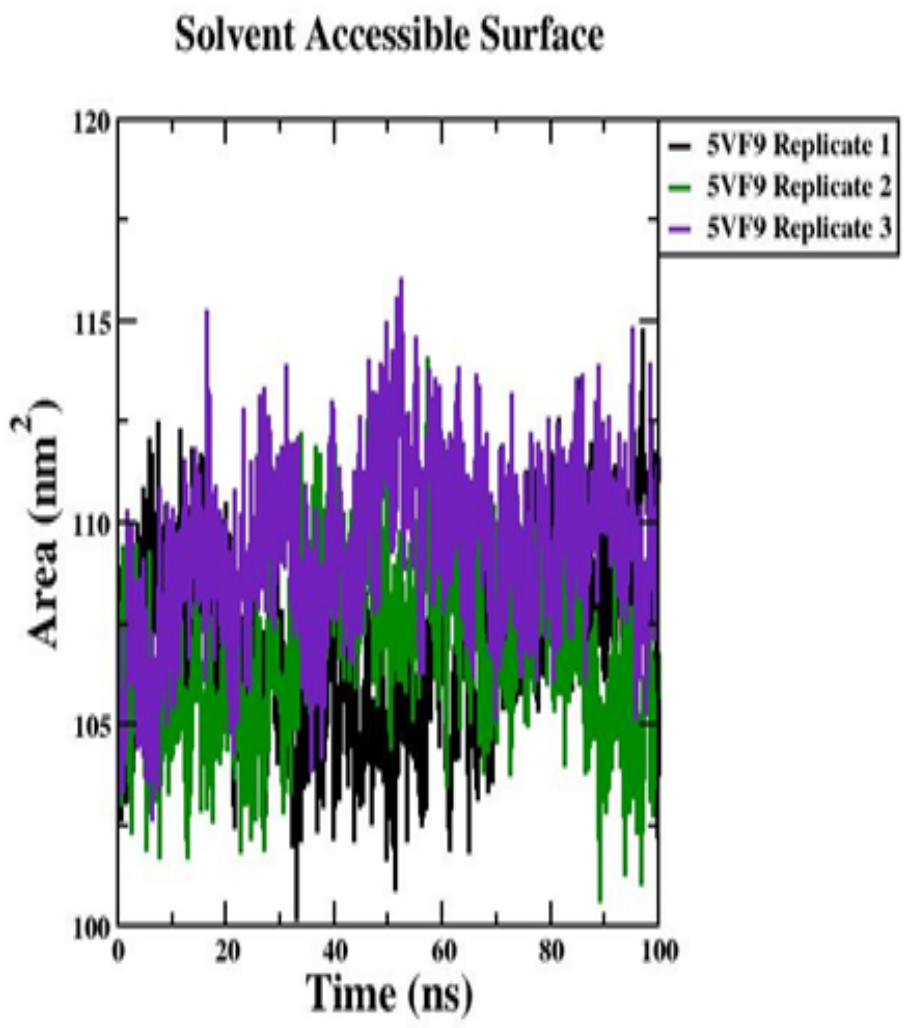


(A) 5VF9 (Triplicate )

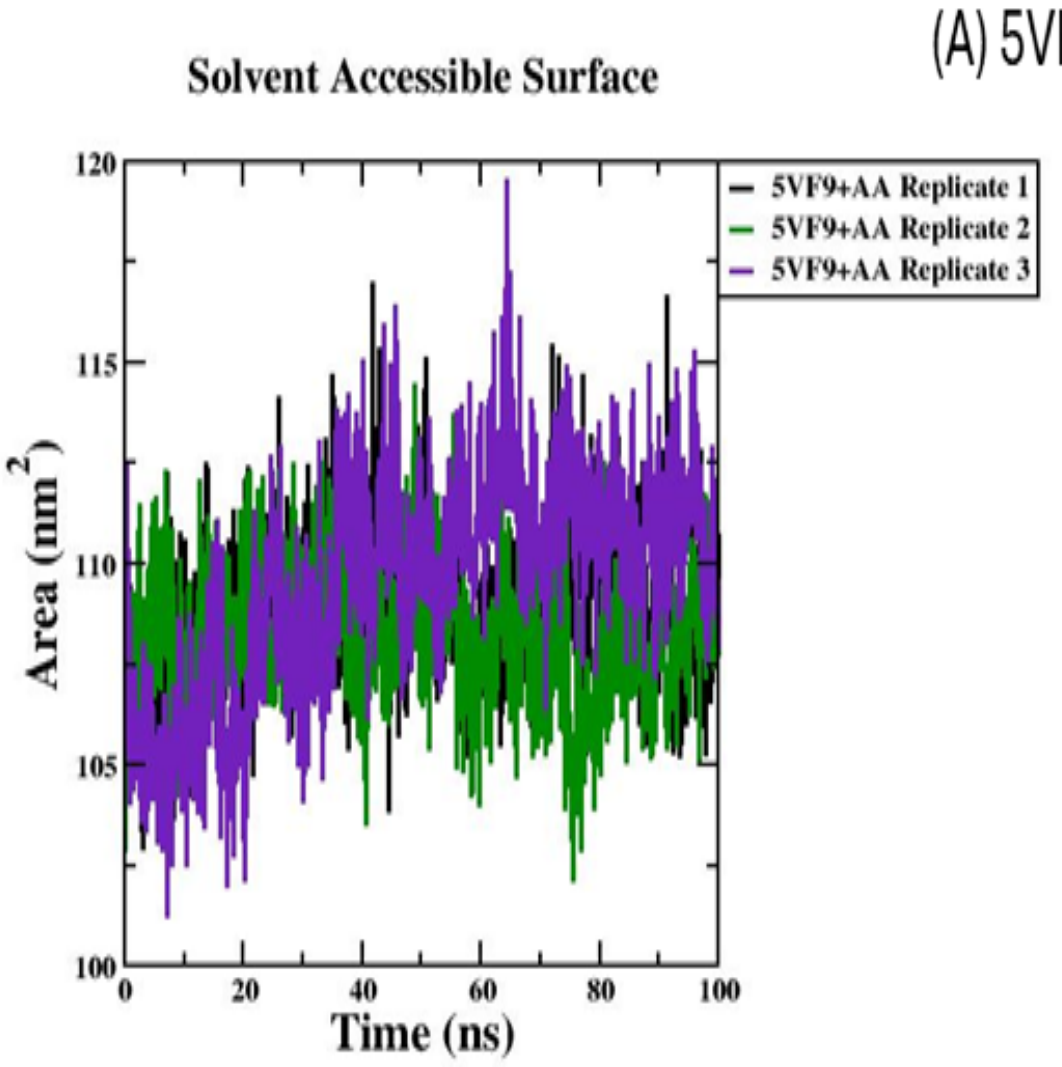


(B) 5VF9_AA (Triplicate )

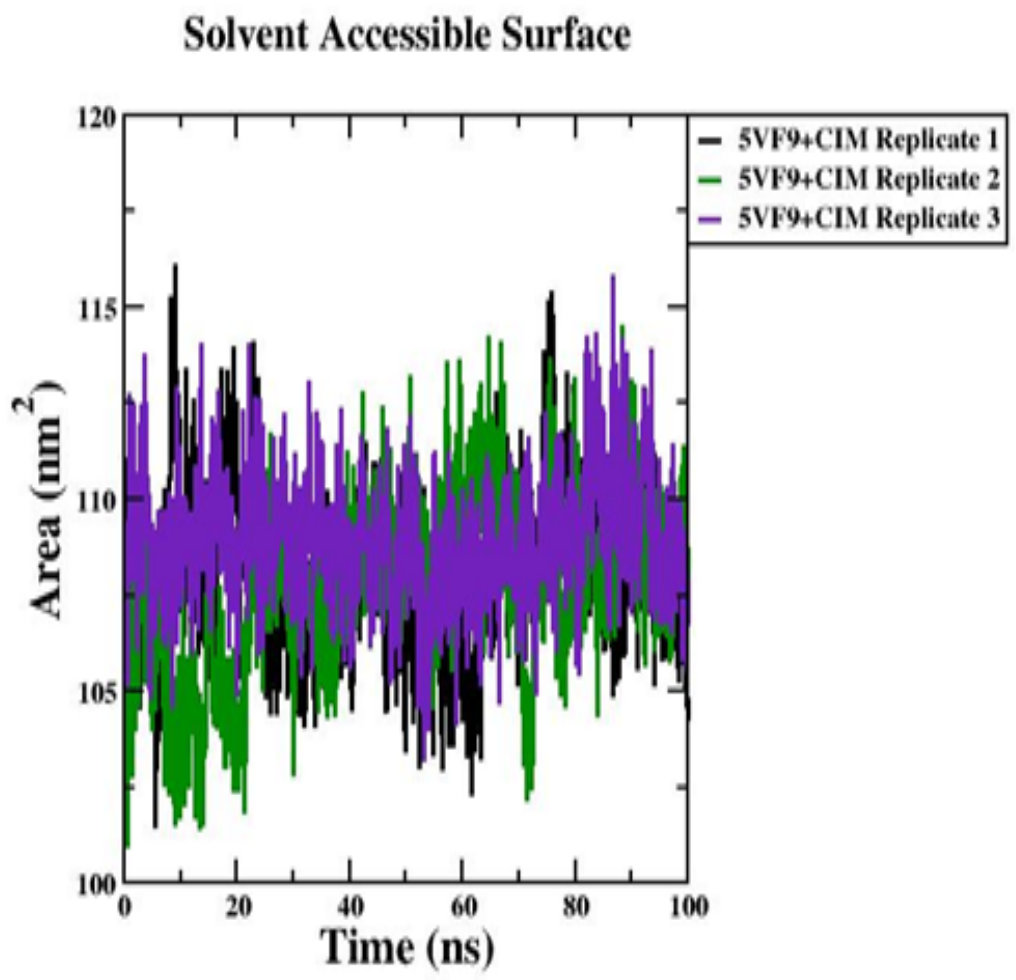


(C) 5VF9_CIM (Triplicate )

**Supplementary Figure S5.** Solvent accessible surface area (SASA) analysis from triplicate 100 ns MD simulations for (A) Apo form of the protein, (B) Ascorbic acid-bound complex, and (C) CIM-bound complex.
