# Supplementary material for: UPLC-MS metabolite profiling and antioxidant efficacy of Neopestalotiopsisclavispora isolated from Oroxylum indicum (L.) Kurz Stem Bark
Source: PLoS One. 2026 Sep 9;21(9):e0357595. doi: 10.1371/journal.pone.0357595 (PMC13557386; doi:10.1371/journal.pone.0357595)
Supplement: Supplementary file 8 [file pone.0357595.s008.pdf]

Supplementary Tables S1 and S2. MM-PBSA-derived binding free energies along with individual energy contribution parameters for the 5VF9–AA and 5VF9–CIM complexes obtained from triplicate molecular dynamics simulations.

**Ascorbic acid Energy contributed (kcal/mol)**					
**Type of energy**	Replicate 1	Replicate 2	Replicate 3	Average	Standard deviation
van der Waal energy	-2.53	-1.92	-2.1	-2.18333	0.255908
Electrostatic energy	-5.34	-3.84	-4.44	-4.54	0.616441
Polar solvation energy	6.55	4.4	5.49	5.48	0.877762
Binding energy	-1.32	-1.35	-1.05	-1.24	0.134907

**Cimicifoetiside B Energy contributed (kcal/mol)**					
**Type of energy**	Replicate 1	Replicate 2	Replicate 3	Average	Standard deviation
van der Waal energy	-19.49	-24.53	-15.09	-19.7033	4.723614
Electrostatic energy	-13.71	-4.8	-8.44	-8.98333	4.479781
Polar solvation energy	19.95	15.23	13.81	16.33	3.214405
Binding energy	-13.26	-14.11	-9.72	-12.3633	2.328311
